# Advanced Solid Lubrication with COK‐47: Mechanistic Insights on the Role of Water and Performance Evaluation

**DOI:** 10.1002/advs.202415268

**Published:** 2025-01-13

**Authors:** Hanglin Li, Xudong Sui, Pablo Ayala, Edoardo Marquis, Hannah Rabl, Adrian Ertl, Pierluigi Bilotto, Yazhuo Shang, Jiusheng Li, Lu Xu, Maria Clelia Righi, Dominik Eder, Carsten Gachot

**Affiliations:** ^1^ Institute for Engineering Design and Product Development Research Unit Tribology E307‐05 TU Wien Vienna 1060 Austria; ^2^ Laboratory for Advanced Lubricating Materials Shanghai Advanced Research Institute Chinese Academy of Sciences Shanghai 201210 China; ^3^ Institute of Materials Chemistry TU Wien Vienna 1060 Austria; ^4^ Department of Physics and Astronomy “Augusto Righi” Alma Mater Studiorum‐University of Bologna Bologna 40127 Italy; ^5^ Key Laboratory for Advanced Materials School of Chemistry and Molecular Engineering East China University of Science and Technology Shanghai 200237 China; ^6^ State Key Laboratory of Solid Lubrication Lanzhou Institute of Chemical Physics Chinese Academy of Sciences Lanzhou 730000 China

**Keywords:** 2D materials, friction, solid lubrication, titanium‐based MOFs, tribochemistry

## Abstract

Metal–organic framework (MOF) nanoparticles have attracted widespread attention as lubrication additives due to their tunable structures and surface effects. However, their solid lubrication properties have been rarely explored. This work introduces the positive role of moisture in solid lubrication in the case of a newly described Ti‐based MOF (COK‐47) powder. COK‐47 achieves an 8.5‐fold friction reduction compared to AISI 304 steel‐on‐steel sliding under room air. In addition, COK‐47 maintains a similarly low coefficient of friction (0.1–0.2) on various counterbodies, including Al_2_O_3_, ZrO_2_, SiC, and Si_3_N_4_. Notably, compared to other widely studied MOFs (ZIF‐8, ZIF‐67) and 2D materials powder (MXene, TMD, rGO), COK‐47 exhibits the lowest friction (≈0.1) under the same experimental settings. Raman spectroscopy, X‐ray diffraction, X‐ray photoelectron spectroscopy, energy dispersive X‐ray spectroscopy, scanning electron microscope, and transmission electron microscopy indicate that the tribofilm is an amorphous film obtained by hydrolysis of COK‐47 in the air with moisture. Density functional theory further confirms that water catalyzes the decomposition of COK‐47, a crucial step in forming the tribofilm. This study demonstrates the idea of utilizing MOF and water‐assisted lubrication mechanisms. It provides new insights into MOF applications in tribology and highlights interdisciplinary contributions of mechanical engineering and chemistry.

## Graphical abstract

This study is the first to report the solid lubrication performance of the newly discovered Ti‐based MOF (COK‐47) powder. COK‐47 exhibits a remarkably lowest and most stable coefficient of friction compared to typical solid lubricants. The lubrication mechanism involves water molecules from the air participating in tribochemical reactions, forming an amorphous tribofilm composed of titanium, oxygen, and carbon.

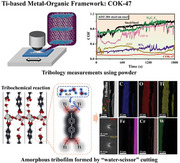

## 1 Introduction

Friction is an unavoidable phenomenon in mechanical systems, causing ≈23% of global energy losses annually due to friction and wear.^[^ ^1^ ^]^ This not only wastes energy and exacerbates environmental pollution but also accelerates component wear, shortens equipment lifespan, and poses safety risks. To address these issues, researchers are developing efficient lubricants to control friction, improve mechanical performance, and extend equipment service life.^[^ ^2^, ^3^, ^4^, ^5^ ^]^

Nanoparticles have recently gained significant attention due to their unique size and surface effects. Among them, metal–organic frameworks (MOFs) are a novel class of nanoparticles formed by coordinating inorganic metal ions or clusters with organic ligands. MOFs feature controllable structures, large specific surface areas, and tunable pore sizes, enabling applications in adsorption and separation,^[^ ^6^, ^7^, ^8^ ^]^ catalysis,^[^ ^9^, ^10^, ^11^ ^]^ energy storage,^[^ ^12^, ^13^, ^14^ ^]^ and drug delivery.^[^ ^15^, ^16^, ^17^ ^]^

In tribology, recent studies highlight the multifunctionality and promising potential as additives. Notably, most research on zeolitic imidazolate frameworks (ZIFs)^[^ ^18^, ^19^, ^20^ ^]^ and University of Oslo (UiO) MOFs^[^ ^21^, ^22^, ^23^ ^]^ have demonstrated significant advantages. Shi et al. first demonstrated that ZIF‐8 and ZIF‐67, as lubrication additives, can enhance the anti‐wear and load‐bearing capabilities of the oil due to their zeolitic structures.^[^ ^20^ ^]^ More and more modified MOF particles have also been discovered to achieve stable dispersion and effective friction‐reducing.^[^ ^24^, ^25^, ^26^ ^]^ Inspired by the excellent lubrication performance of 2D materials,^[^ ^27^, ^28^, ^29^ ^]^ researchers have also developed 2D layered MOFs, including zinc‐based,^[^ ^30^, ^31^, ^32^ ^]^ manganese‐based,^[^ ^33^ ^]^ and copper‐based^[^ ^34^, ^35^ ^]^ variants. They showed synergistic lubrication when used as liquid additives. Zhu et al. further designed a MOF‐on‐MOF heterostructure, where the combination of 0D ZIF‐7 and 2D Ni‐MOF reduced friction.^[^ ^36^ ^]^

The aforementioned studies primarily focus on using MOFs as liquid additives. In the context of non‐liquid lubrication, existing research has largely explored MOFs as enhancement additives in polymer matrices^[^ ^37^, ^38^ ^]^ or hydrogels.^[^ ^39^, ^40^ ^]^ Zhang et al. also utilized MOFs as nanocontainers for lubricants, enabling self‐lubricating coatings.^[^ ^41^ ^]^ However, there are very few studies on the solid lubrication behavior of MOF itself. Moreover, the limited stability of MOFs has remained a major challenge since their discovery.^[^ ^42^ ^]^ Hydrolysis is always playing a negative role in MOFs. For instance, ZIF‐8 will hydrolyze when used in aqueous lubrication, thus hindering sliding. The authors used a polydopamine (PDA) surface modification strategy to inhibit hydrolysis of ZIF‐8.^[^ ^24^, ^26^ ^]^ Notably, it remains unclear whether hydrolysis occurs under solid lubrication conditions, what positive effects it may have on tribological performance, or the mechanistic role of water in MOF tribology.

Herein, this work first investigated the solid lubrication behavior of a new Ti‐based MOF powder. The material was size‐isotropic and defect‐free prepared, named COK‐47_ISO_ (hereinafter referred to as COK‐47 for simplicity).^[^ ^43^ ^]^ COK‐47 is built up from extended 2D secondary building units (SBUs) based on Ti‐O_6_ octahedra, which are interconnected by 4,4″‐biphenyldicarboxylate to form a layered titanium oxide MOF. Friction tests showed that the coefficient of friction (COF) and wear rate using COK‐47 powder decreased by over 88% and 60%, respectively, compared with blank steel‐on‐steel sliding. COK‐47 also showed stable lubrication under different counterbody‐on‐steel sliding and the best lubrication performance compared to other widely studied MOFs or 2D materials used in solid lubrication. The moisture in the air can influence the formation of tribofilm by participating in tribochemical reactions. Raman spectroscopy, scanning electron microscopy (SEM), energy disperse spectroscopy (EDS), X‐ray photoelectron spectroscopy (XPS), X‐ray diffraction (XRD), and transmission electron microscopy (TEM) provided evidence of the tribofilm's structural and chemical composition. Density functional theory (DFT) results showed that water can release internal stresses in COK‐47 by actively participating in tribochemical reactions. This leads to easier bond breaking between atoms within the crystal structure and facilitates the formation of a lubricating film, reducing friction. This study demonstrates the positive role of water in MOF‐based solid lubrication and explores humidity‐assisted lubrication mechanisms. It offers new insights into the application of MOFs in tribology and highlights the interdisciplinary contributions of mechanical engineering and chemistry.

## 2 Results and Discussion

### 2.1 Structure and Chemical Composition

**Figure 1:**
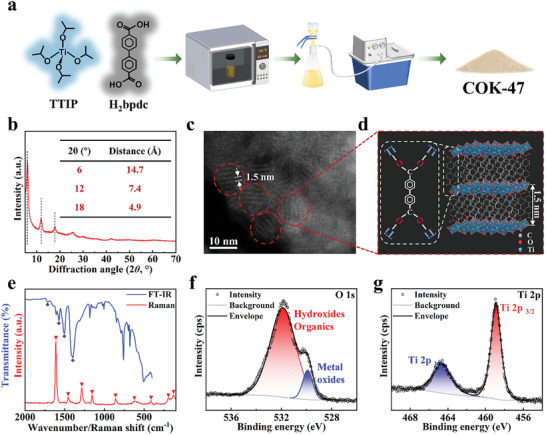
a) The preparation process of COK‐47 by microwave heating. b) XRD results of COK‐47. c) HAADF TEM image and d) structural diagram of COK‐47. The particles are marked in red dashed lines, and the layer distance is ≈1.5 nm. e) FT‐IR and Raman spectra, detailed XPS spectra of f) O 1s and g) Ti 2p.

MOFs are usually built up from organic ligands and 0D clusters. Higher dimensionality (1D rods or 2D sheets) SBUs have recently been described.^[^ ^44^ ^]^ Based on a recent study,^[^ ^43^ ^]^ COK‐47, a Ti‐based 2D SBUs MOF, was synthesized using an optimized microwave‐assisted synthesis procedure (**Figure** **1**a). This process quickly yields small, size isotropic, and defect‐free particles at moderate temperatures. The characterization results of COK‐47 are displayed in Figure 1b–g. In Figure 1b, the pronounced crystallographic signals attributable to the COK‐47 structure were observed. From the wide reflexes observed in the XRD results, a high degree of disorder in COK‐47 can be confirmed, which is attributed to its small crystallites with no preferential orientation. The TEM image in Figure 1c showed small nanocrystals, ≈9–10 nm in size. The distance among the layers in the nanocrystal was uniform and ≈1.5 nm, consistent with the organic linker's size and the results from the literature.^[^ ^43^, ^45^ ^]^ Titanium oxide layers are connected by titanium‐carboxylate coordination, forming a MOF with 2D SBUs (Figure 1d).

For chemical composition, the blue marks from FT‐IR in Figure 1e showed the characteristic peaks of COK‐47 in 1714, 1573, 1506, and 1402 cm^−1^, which aligns well with our previous research.^[^ ^38^ ^]^ The Raman spectrum exhibited characteristic peaks at 1611, 1456, 1285, 1156, and 859 cm^−1^, which correspond to the vibrational signals of strong aromatic ring stretching, weak aromatic ring stretching, inter‐aromatic ring stretching, weak in‐plane deformation, and weak out‐of‐plane deformation, respectively. XPS analysis of the survey spectrum (Figure S1, Supporting Information) revealed the elemental composition of Ti:O:C = 1:3.2:7.9 in COK‐47, which goes well in line with the theoretical ratios in the molecular formula of Ti_2_O_3_(BPDC), i.e., Ti:O:C = 1:4:7. The O 1s spectrum (Figure 1f) showed the presence of two oxygen species, corresponding to metal–oxygen interactions (Ti–O–Ti) of the SBU, as well as oxygen originating from the ligand's carboxylic groups. The Ti 2p spectrum suggested the oxidation state to be +IV (Figure 1g), which aligns with previous results from the reference. In summary, these results proved the successful preparation of COK‐47.

### 2.2 Tribological Properties

**Figure 2:**
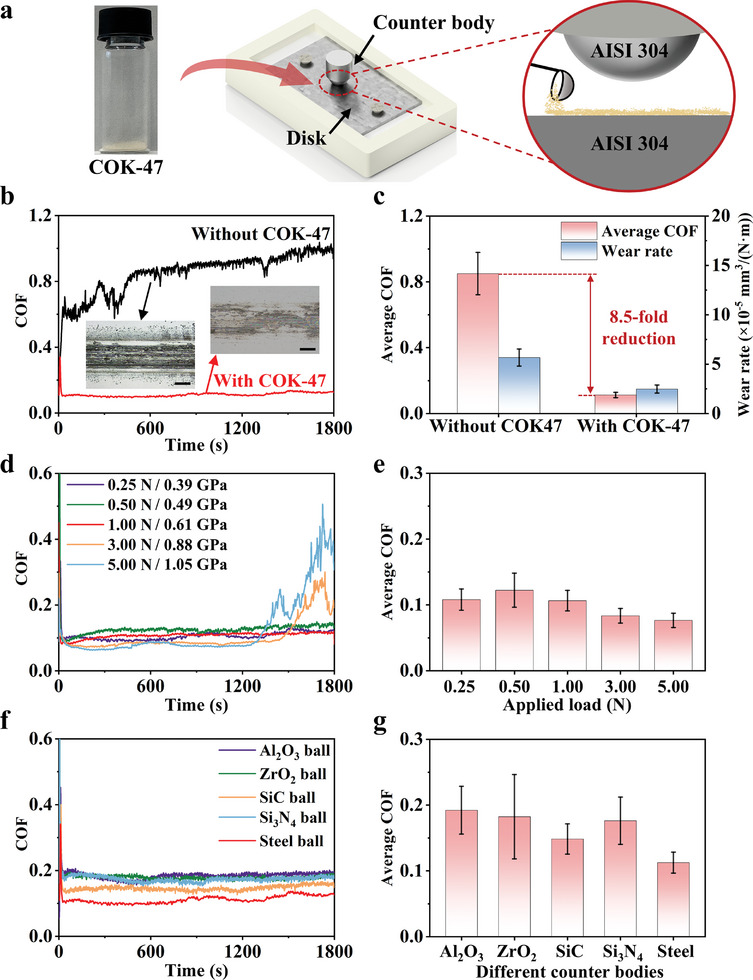
Tribological properties of COK‐47. a) The friction measurements were conducted using a simple as‐synthesized powder on a ball‐on‐disk tribometer. b) COF curves versus time without and with COK‐47. The insets are optical images of the wear track after the test. The scale bars are both 100 µm. The experimental parameters are as follows: 0.25 N, 1 mm s^−1^, AISI 304 ball, AISI 304 disk, room temperature (RT), and air with ≈40% relative humidity (40% RH air). c) Average COF and wear rate comparison between without and with COK‐47. COK‐47 shows an 8.5‐fold friction reduction compared to AISI 304 steel‐on‐steel sliding. d) COF versus time with COK‐47 under 0.25, 0.50, 1.00, 3.00, and 5.00 N loads. e) Average COF over the stable period for the curves in Figure 2d. The other experimental parameters are as follows: 1 mm s^−1^, AISI 304 ball, AISI 304 disk, RT, and 40% RH air. f) COF of COK‐47 versus time under under Al_2_O_3_, ZrO_2_, SiC, Si_3_N_4_, and steel counterbodies. e) Average COF of the curves in Figure 2f (0.25 N, 1 mm s^−1^, AISI 304 disk, RT, 40% RH air).

The friction tests of COK‐47 powder were conducted using the method shown in **Figure** **2**a. Figure 2b,c shows the tribological performance with and without COK‐47 lubrication. It was found that under ambient room temperature conditions, COK‐47 exhibited excellent and stable lubrication performance, reducing the COF and wear rate between steel surfaces by 8.5 and 2.3 times, respectively. The inset wear track images show that COK‐47 lubrication made the wear track surface smoother, and a large area of colorful tribofilms was observed. In contrast, without COK‐47 lubrication, the wear track on the steel surface appeared rougher and displayed black debris morphology. The lower roughness of the wear track with COK‐47 lubrication was further confirmed in (Figure S2, Supporting Information).

To further investigate the load‐carrying capacity of COK‐47, friction tests were conducted under different loads (contact pressures are 0.39, 0.49, 0.61, 0.88, and 1.05 GPa) as shown in Figure 2d,e. Under low loads (0.25, 0.50, and 1.00 N), COK‐47 provided a stable and low COF. When the load was increased to 3.00 and 5.00 N, the COF curves remained relatively stable initially but exhibited significant fluctuations at ≈1500 and 1300 s, respectively (Figure 2d). This suggests that the tribofilm formed during friction is consumed more rapidly at higher contact pressures, leading to direct contact between the frictional components. However, even after high‐load testing, residual colorful films were still observed at the boundaries between the wear tracks and the untouched substrate (Figure S3, Supporting Information). Therefore, it can be inferred that COK‐47 achieves its superior friction‐reducing performance by forming colorful tribofilms during sliding. As the load and time increase, these tribofilms are gradually consumed, resulting in a significant rise in COF.

In addition, this study also investigated the friction performance of COK‐47 under various counterbody conditions (Figure S4, Supporting Information for more details). The friction results (Figure 2f,g) indicate that COK‐47 exhibited consistently low COF values (≈0.1–0.2) across different counterbodies. It highlights the advantageous substrate‐independent property as a solid lubricant and suggests that COK‐47 may be suitable for various lubrication scenarios.

### 2.3 Lubrication Mechanism

Friction results suggested that enhanced lubrication performance is associated with more pronounced colorful tribofilms in the contact area. To gain deeper insights, it is essential to investigate the composition, structure, and formation mechanism of tribofilm.

#### 2.3.1 Exploring the Formation of Tribofilm from an Experimental Perspective

**Figure 3:**
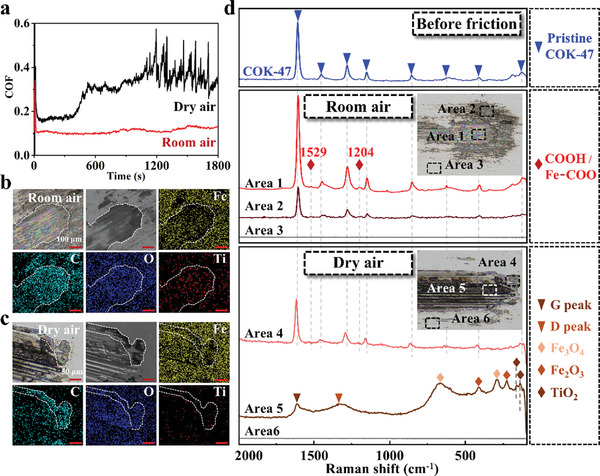
a) COF versus time under dry air and room air. The test conditions are 0.25 N, 1 mm s^−1^, AISI 304 ball, AISI 304 disk, RT. Panels (b) and (c) are the corresponding SEM and EDS mapping results of the wear track obtained from **Figure** **3**a. Panel (b) for room air and panel (c) for dry air. The EDS signal of iron (Fe), carbon (C), oxide (O), and titanium (Ti) are represented by yellow, cyan, blue, and red, respectively. d) Raman analysis of different areas on the wear tracks after friction under room air and dry air. Insets are the optical images of the wear track.

**Figure** **3**a shows the COF curves of COK‐47 under room air (≈40% RH) and dry air (less than 10% RH) conditions. In dry air, COK‐47 exhibited a higher COF and an unstable curve over time. In contrast, the COF curve remained lower and more stable under room air conditions. Comparing the wear track images (Figure 3b,c), it can be found that the colorful tribofilm had more impact and covered the entire area of the wear track under room air. In contrast, only a small amount of colorful tribofilm was observed under dry air conditions at the edges of the wear track. EDS mapping results showed that the colorful film region was mainly composed of C, O, and Ti elements, while the wear track under dry air conditions contained more Fe and O elements. The specific elemental ratios in different regions of the wear track can be found in Figure S5 and Table S1 (Supporting Information).

Raman analysis was performed to investigate the chemical group variations (Figure 3d). When comparing pristine COK‐47 (blue line) with the colored tribofilm formed under room air (Area 1), two new Raman peaks were observed in the tribofilm at 1529 and 1204 cm^−1^, corresponding to ─COOH or Fe─COO coordination bonds.^[^ ^46^ ^]^ The appearance of these groups suggested that the tribofilm likely originated from the hydrolysis of COK‐47. The Raman peaks in the wear track boundary (Area 2) were identical to those of pristine COK‐47, as this region was not subjected to full frictional stress. A slight red shift was observed in Area 2, attributed to water adsorption.^[^ ^47^ ^]^ This indicates that the formation of the tribofilm requires the combined effects of hydrolysis and frictional force.

In dry air, Area 4 showed peaks at 1611, 1456, 1285, 1156, 859, and 629 cm^−1^, consistent with pristine COK‐47, but no peaks induced by frictional hydrolysis were observed. Besides, the signal from Area 4 showed blue shifts due to the loss of physisorbed water.^[^ ^47^ ^]^ In the dark area of the wear track (Area 5), Raman signals revealed iron oxides (Fe_2_O_3_: 412, 227 cm^−1^; Fe_3_O_4_: 669, 292 cm^−1^),^[^ ^48^ ^]^ titanium oxides (TiO₂: 161, 140 cm^−1^),^[^ ^49^, ^50^ ^]^ and carbon peaks (D: 1338 cm^−1^, G: 1613 cm^−1^),^[^ ^51^ ^]^ indicating COK‐47 underwent carbonization and decomposition in this area. All Raman signals are summarized in Table S2 (Supporting Information).

Overall, Raman analysis indicated that under room air, COK‐47 underwent hydrolysis and formed tribofilm by tribochemical reactions. In contrast, under dry air, COK‐47 formed distinct carbon films and metal oxide tribofilm under friction, resulting in a higher COF. These findings suggest that water may promote tribochemical reactions involved in tribofilm formation. Additional friction tests under different humidity conditions (Figure S6, Supporting Information) also confirmed that water can influence the formation of the tribofilm. The colored tribofilm was difficult to form under low humidity conditions (7.7% to 20.5%), while the most complete and abundant tribofilm formed when the humidity was ≈40%. As the humidity continued to increase to 83.5%, the colored tribofilm was still effectively formed and covered the inside region of the wear track.

**Figure 4:**
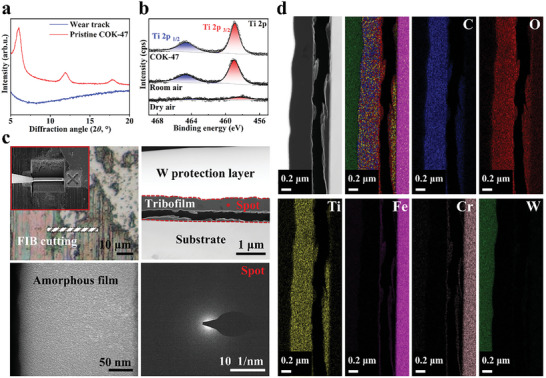
Characterization of tribofilm. a) XRD results from the wear track and COK‐47 before friction. b) Ti 2p XPS spectra analysis on the wear tracks under room and dry air environmental conditions. c) FIB cutting preparation and morphology of the colorful tribofilm come from the wear track after the measurement (0.25 N, 1 mm s^−1^, RT, and 40% RH air). The striped frame was the area for FIB cutting. The inset was the enlarged view. TEM images and SAED pattern indicated that the tribofilm is amorphous. d) EDS mapping results of the corresponding tribofilm.

A more detailed structure and composition analysis was conducted on the colored tribofilm within the wear track as shown in Figure 4. The XRD results (Figure 4a) demonstrated that pristine COK‐47 exhibited crystalline reflection peaks in the 2*θ* range of 4–20°, whereas no corresponding diffraction peaks were observed in the tribofilm of COK‐47. This indicated the amorphous structure of the tribofilm. Additionally, the XPS Ti 2p spectrum analysis in Figure 4b confirmed that titanium remained in the +IV oxidation state under pristine COK‐47, dry air, or room air after friction. Based on the results from XRD and XPS, it is indicated that the organic linkers between the Ti‐O layers were probably disrupted, resulting in a disordered structure. However, the Ti 2p signal in XPS results under dry air was much weaker in room air (Figure 4b). This highlights the significant impact of water molecules on the formation of tribofilm and suggests a potential hydrolysis mechanism.

A focused ion beam (FIB) cross‐section was prepared from a representative wear track under room air for TEM analysis (Figure 4c). The FIB cutting process is shown in Figure S7 (Supporting Information). The cross‐sectional morphology revealed a smooth and dense tribofilm with a thickness of ≈240 nm. High‐resolution TEM images showed no crystalline structure, and the selected area electron diffraction (SAED) patterns also displayed a characteristic amorphous halo (Figure 4c). This indicated that COK‐47 underwent amorphization during friction and formed a dense, disordered distribution of small molecular fragments. Energy‐dispersive X‐ray spectroscopy (EDS) mapping (Figure 4d) showed the tribofilm was composed of C, O, and Ti. There was a uniform distribution of these elements within the tribofilm. The linear elemental scanning results (Figure S8, Supporting Information) also supported this finding. The atomic ratios of every element are shown in Table S3 (Supporting Information). The calculated Ti: O: C atomic ratio (1:4.77:7.22) closely matches the theoretical ratio (1:4:7) of pristine COK‐47. Some of the “extra” oxygen atoms may come from water involved in tribochemical reactions. This dense, polymer‐like amorphous tribofilm effectively reduced surface direct contact friction. These results explain why COK‐47 powder can exhibit excellent tribological performance.

#### 2.3.2 Exploring the Formation of Tribofilm from a Molecular Simulation Perspective

**Figure 5:**
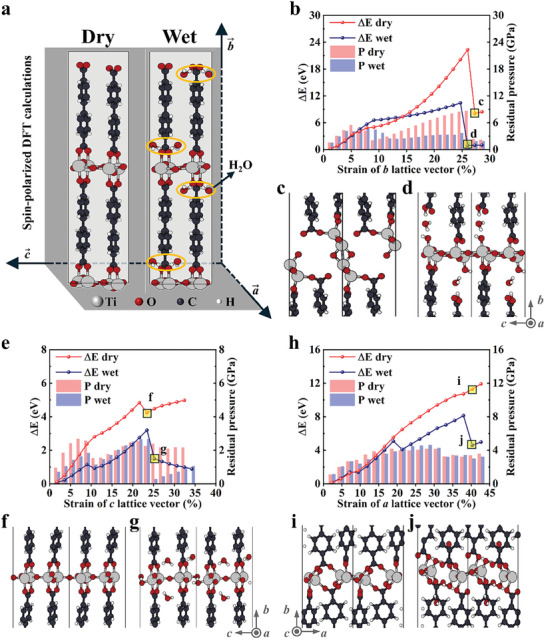
DFT simulations. a) The unit cells used for dry and wet COK‐47. The orange circle areas refer to water molecules. b) Energy cost (left axis) and residual pressure (right axis) experienced during the uniaxial strain of the *b* lattice vector, for dry‐ (red) and wet‐ (blue) COK‐47. The structural features of the dissociation products are shown in panels (c) and (d) for dry‐ and wet COK‐47 respectively. The atomic color is as follows: Ti (gray), O (red), C (black), H (white). In terms of other lattice vectors, energy cost (left axis) and residual pressure (right axis) are experienced during the uniaxial strain along the panel (e) *c* and (h) *a* lattice vectors. Red (blue) curves and bars refer to dry‐ (wet) COK‐47. The main structural features of dissociation products along the *c* lattice vector are shown in panels (f) and (g) for dry and wet COK‐47, respectively, while those along *a* lattice vector are shown in panels (i) and (j).

The experimental results demonstrated that atmospheric water plays a crucial role in the tribological performance of COK‐47 powder. The formation of tribofilm relies on atmospheric humidity. Density functional theory (DFT) calculations were performed to elucidate the specific role of water in the decomposition of COK‐47. The unit cells used in the simulations (**Figure** **5**a) represented “dry” and “wet” COK‐47. The “wet” model incorporated 8 water molecules into the bulk of dry COK‐47. Hydration of COK‐47 was found to be thermodynamically favorable, with an energy gain of 0.11 eV per water molecule. This process was facilitated by stable hydrogen bonds formed between water and the oxygen atoms of the aromatic ligands.

The lattice vectors (*a*, *b*, and *c*) were incrementally stretched from the relaxed structures to simulate the shear effect on randomly oriented COK‐47 particles. Results for *b* vector, aligned parallel to the aromatic ligands, are shown in Figure 5b. The corresponding results for the *a* and *c* vectors are presented in Figure 5e,h. The energy required for stretching (ΔE, left axis) and the residual pressure on the unit cell (right axis) were recorded. For strains below 8%, both dry (red curve in Figure 5b) and wet (blue curve in Figure 5b) COK‐47 exhibited a similar elastic response. This is attributed to a slight length increase of the Ti─O bonds aligned along the *b* vector. In the early stretching stages, the presence of water does not attenuate the increase in energy.

However, differences emerged for strains from 8% to 25%. Wet COK‐47 accommodated deformations more effectively, as indicated by the reduced slope of the blue curve. For strains beyond 25%, the internal atomic arrangement of COK‐47 started to collapse, releasing accumulated energy. While decomposition occurred in both cases, wet COK‐47 has a much lower energy cost due to water molecules catalyzing the dissociation of COO‐Ti bonds. The dissociation process (Figure 5c for dry COK‐47 and Figure 5d for wet COK‐47) confirmed the active role of water, which hydrolyzed the strained COO─Ti bond into COOH and HO─Ti.

The shaded bars in Figure 5b showed the residual pressure acting on the unit cell during stretching. Higher residual pressure corresponds to greater resistance to deformation and lower stress accommodation. Wet COK‐47 exhibited a maximum residual pressure of 5.0 GPa, significantly lower than the 8.7 GPa for dry COK‐47. This indicated that water molecules facilitate deformations by forming H‐bonds with carboxyl groups and donating electron density to the undercoordinated Ti atoms.

Similar trends were observed when stretching along *a* (Figure 5e) and *c* (Figure 5h) lattice vectors. These DFT results suggested that environmental water molecules actively drive the formation of an amorphous tribofilm. They enable internal stress release by participating in tribochemical reactions such as H‐bonds formation, hydrolysis, and passivation of Ti dangling bonds. It is hypothesized that COK‐47′s lubricant ability depends on structural deformations, which are significantly facilitated by the presence of moisture.

### 2.4 Comparison and Application Prospect

**Figure 6:**
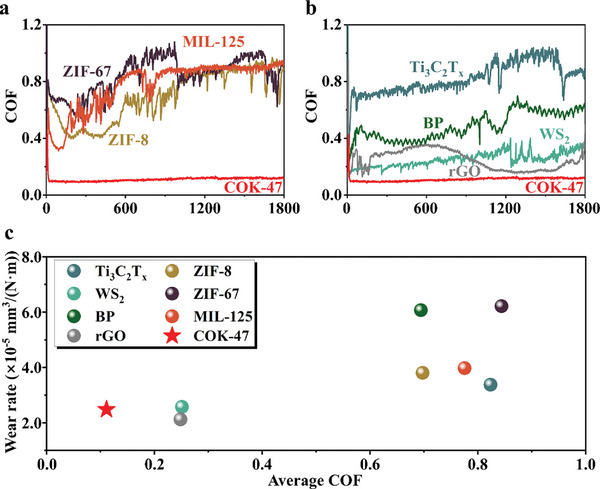
Comparison of COK‐47 with other lubrication materials. a) COF curves of COK‐47 and other MOFs, including the most widely studied ZIF family (ZIF‐8 and ZIF‐67) and Ti‐based MOF (MIL‐125). b) COF curves of COK‐47 and other widely studied 2D materials (Ti_3_C_2_T_x_, WS_2_, BP, rGO). Among the curves, COK‐47 showed the best performance. c) Comparison of tribological performance (average COF and wear rate) between COK‐47 and other widely studied materials.

To evaluate the superior performance of COK‐47, it was compared to other widely studied materials. Zeolite imidazolate framework (ZIF) family is the most widely studied MOF in tribology.^[^ ^52^ ^]^ MIL‐125 is a Ti‐based MOF from matérial institut lavoisier (MIL) family. In addition, 2D materials in tribology including MXene family (Ti_3_C_2_T_x_), transition metal dichalcogenide (TMD) family (WS_2_), black phosphorus (BP), and graphene family (reduced graphene oxide, rGO) were selected to competitive tests. The results are shown in **Figure** **6**. COK‐47 exhibited the most stable and lowest COF curve, both compared to other MOFs (Figure 6a) and to 2D materials (Figure 6b). In terms of the promising solid lubricant candidate, not only the value of COF but also the wear rate should be considered. Figure 6c shows the results of average COF and wear rate obtained from COK‐47 and different representative material powders as described before under steel‐on‐steel sliding. The other experimental parameters are as follows: 0.25 N (0.39 GPa), 1 mm s^−1^, stroke of 1 mm, RT, and 40% RH room air. Specifically, COK‐47 powder demonstrated the lowest average COF of ≈0.1 and a wear rate as low as 2.4 × 10^−5^ mm^3^/(N·m) during sliding against steel under atmospheric conditions. Besides, friction tests under alumina‐on‐steel sliding were also conducted (Figure S9, Supporting Information). The results showed that COK‐47 exhibited the most superior lubrication performance when sliding against either steel or alumina counterbodies, which means that COK‐47 could be a promising candidate in solid lubrication.

The lubrication performance of COK‐47 in this study was also compared with others in previous publications (Figure S10, Supporting Information). The presented statistics focus exclusively on atmospheric conditions and highlight 2D solid lubricants, including MXenes,^[^ ^53^, ^54^ ^]^ TMDs,^[^ ^55^, ^56^, ^57^, ^58^ ^]^ GO^[^ ^59^ ^]^ and their composite coatings. It is worth noting that COK‐47 powder's performance is comparable to some of the coatings, which even surpasses that of some coatings. Many researchers have reported that preparing the powder to the coating can significantly improve tribological performance.^[^ ^60^, ^61^, ^62^ ^]^ This also implies that the tribological performance of COK‐47 is expected to be further enhanced if it is prepared as coatings or thin films by suitable methods. Besides, surface texturing on sliding partners is another strategy to improve the retainment of solid lubricant in the contact zone.^[^ ^54^ ^]^ It may help COK‐47 powder be more effectively retained at contact points during prolonged use or dynamic conditions. Therefore, COK‐47 shows the best lubrication performance among commonly used powders and holds strong potential for further improvement. These findings indicated that COK‐47 is a potential candidate for future solid lubrication.

## 3 Conclusion

In summary, this work reported for the first time the solid lubrication behavior of a newly reported Ti‐based MOF, COK‐47. COK‐47 powder can achieve a low COF (≈0.1) in 40% RH room air during AISI 304 steel‐on‐steel sliding. In addition, COK‐47 demonstrated significant friction reduction when applied with different counterbodies such as Al_2_O_3_, ZrO_2_, SiC, and Si_3_N_4_. The counterbody‐independent property suggests a promising potential for solid lubrication applications. COK‐47 outperformed other commonly used MOFs (MIL‐125, ZIF‐8, ZIF‐67) and 2D materials (MXenes, TMDs, rGO, BP) in solid lubricant filed under the same experimental conditions. Spectroscopic methods (Raman, XRD, XPS, EDS) and electron microscopes (SEM and TEM) confirmed the occurrence of tribochemical reactions during friction in 40% RH air. It was also found that the tribofilm is a 240 nm‐thick amorphous layer composed of titanium, oxygen, and carbon. Finally, DFT simulation further indicated that water can participate in the cleavage of Ti‐O bonds in Ti‐O‐C, leading to the formation of tribofilm. In the future, further studies of COK‐47′s performance under extreme conditions, such as high loads or elevated temperatures, will be explored. Additionally, developing COK‐47 coatings through spraying or other methods holds promise for extending service life. This work can highlight the positive role of water molecules in the MOF solid lubrication and provide a new view to the application of MOFs in tribology.

## 4 Experimental Section

### Materials

4′4‐Biphenyl dicarboxylic acid (H_2_bpdc, C_14_H_10_O_4_, 98.0%, ABCR) was used as the ligand, and titanium tetraisopropoxide (TTIP, [OCH(CH_3_)_2_]_4_Ti, 97.0%, Sigma‐Aldrich) was used as the Ti precursor. Anhydrous dimethylformamide (DMF, C_3_H_7_NO, 99.8%, Acros Organics) and HPLC grade methanol (MeOH, CH_3_OH, 99.8%, VWR) were used as a solvent for preparing and washing COK‐47. All the chemicals were used as received. The sources for other materials mentioned in this paper are as follows: Ti_3_C_2_T_x_ was obtained by the etching process, as shown in previous research.^[^ ^53^ ^]^ WS_2_ obtained by Tribotecc, Austria. RGO was prepared by the reduction of graphene oxide using _L_‐Ascorbic acid.^[^ ^63^, ^64^ ^]^ The high‐purity black phosphorus (BP) super‐saturated solution (≈200 mg L^−1^, lateral size: 100 nm‐5 µm, thickness: 1–10 layers, purity 99.998%) was purchased from the company (Smart‐elements GmbH, Austria). The BP powder was obtained by solvent‐evaporation method. MIL‐125 was synthesized using solvothermal method.^[^ ^43^ ^]^ ZIF‐8 and ZIF‐67 were synthesized using Zn^2+^ and Co^2+^ with 2‐methylimidazole as in previous work.^[^ ^65^ ^]^

### Synthesis of COK‐47 Powder

COK‐47 was prepared according to the optimized method for size‐isotropic, defect‐free particles reported by a recent study.^[^ ^43^ ^]^ All operations were performed under an inert Ar atmosphere. H_2_bpdc (242 mg, 1 mmol) was suspended in a mixture of anhydrous DMF (9 mL) and HPLC MeOH (1 mL) and subjected to sonication treatment for 5 min. After adding TTIP (148 µL, 0.5 mmol) to the H_2_bpdc suspension, the mixture was sonicated for 1 min, followed by microwave‐assisted heating (Monowave 300, Anton Paar) for 60 min at 150 °C with 600 rpm stirring. After the reaction, the mixture was transferred into a vacuum filtration apparatus to remove unreacted reagents. The sample was washed 3 times with DMF and MeOH, respectively. Then, the product was pre‐dried for 30 min at 60 °C. Afterward, it was ground to powder and dried overnight at 150 °C under vacuum.

### Friction Tests

Friction tests were performed on a ball‐on‐disk linear reciprocating machine (Rtec, USA). The stainless‐steel 304 ball (AISI 304) with a 6.35 mm diameter was used as the counterbody. The disk was bright‐polished stainless‐steel 304 (AISI 304, 10 mm × 10 mm × 1 mm). For the measurements under different counterbodies, the Al_2_O_3_, ZrO_2_, SiC, and Si_3_N_4_ balls are all 6.35 mm in diameter. Before testing, the protective film was removed, and the disk was sonicated for 5 min in isopropanol and ethanol, respectively, followed by blow‐drying. COK‐47 (5 mg) powder was put on the substrate for each test. The sliding stroke was 1 mm. In different normal load tests, the loads were set to 0.25, 0.50, 1.00, 3.00, and 5.00 N (the calculated contact pressures are 0.39, 0.49, 0.61, 0.88, and 1.05 GPa, respectively), and the sliding velocity was 1 mm s^−1^. The airtight chamber was used to stabilize the humidity during the test. Before every test, the relative humidity was stabilized for at least 10 min. Each test was repeated 3 times to ensure data availability. All tests were performed at a temperature of 24–26 °C.

### Materials Characterization Methods

To determine the structure and composition of COK‐47 powder, several techniques were used, including Attenuated‐Total Reflection Fourier‐Transformed Infrared Spectroscopy (ATR‐FTIR), Raman spectroscopy, X‐ray diffraction (XRD), X‐ray photoelectron spectroscopy (XPS), and high‐angle annular dark field scanning transmission electron microscopy (HAADF‐STEM). ATR‐FTIR analysis was performed using a Bruker Tensor 27 spectrometer (Ettlingen, Germany) equipped with a diamond crystal accessory, applied in the solid state for all samples. Raman spectroscopy was performed on a LabRam Aramis Raman microscope (Horiba Jovin Yvon, Germany). The samples were excited using a laser wavelength of 532 nm at a laser power of 2 mW. XRD analysis was carried out using an XPERT II: PANalytical XPert Pro MPD (Θ–Θ Diffractometer). The sample was positioned on a silicon holder and exposed to Cu Kα radiation (8.04 keV and 1.5406 Å). Data were collected over a 5–70° range using Bragg–Brentano Θ/Θ‐diffractometer geometry with a semiconductor X'Celerator detector (2.1°). XPS test was obtained using a custom‐built SPECS XPS spectrometer, which featured a monochromatic Al‐Kα X‐ray source (µFocus 350) and a hemispherical WAL‐150 analyzer. The operating conditions were as follows: excitation energy of 1486.6 eV; beam energy and spot size of 70 W with a 400 µm focus; measurement angle of 51° relative to the sample surface normal; base pressure of 5 × 10^−10^ mbar; and measurement pressure of 6 × 10^−9^ mbar. Survey spectra were recorded at pass energies of 100 eV, while for detailed spectra pass energies of 30 eV were used. Charge correction was applied using the C 1s peak for adventitious carbon, shifting it to 284.8 eV binding energy (BE) according to Biesinger et al.^[^ ^66^ ^]^ XPS data treatment was performed using CasaXPS software and Scofield sensitivity factors.^[^ ^67^ ^]^ Transmission corrections (as per the instrument vendor's specifications) were employed. The C and O Shirley backgrounds^[^ ^68^ ^]^ were employed, and a Shirley Tougaard^[^ ^69^ ^]^ background was used for Ti. High‐angle annular dark field (HAADF) STEM imaging was carried out using a Titan Cubed G2 60–300 (TEM/STEM, FEI Co., now Thermo Fisher Scientific) at an operating voltage of 300 kV. This microscope is equipped with an aberration corrector for STEM (DCOR, CEOS) and a four‐quadrant windowless super‐X SDD system. The probe current for STEM imaging was ≈60 pA, with a convergence semi‐angle of 18 mrad and a typical probe diameter of less than 0.1 nm. Forward scattered electrons, detected by a HAADF detector, were collected within an angular range of 38 to 184 mrad.

### Wear Track Characterization Methods

The wear surfaces were analyzed by laser scanning microscopy (CLSM, Keyence VK‐X1100), Raman spectroscopy, XRD, XPS, SEM‐EDS, and FIB‐TEM. The Raman spectroscopy testing conditions for the wear tracks were consistent with those used for testing the COK‐47 powder. XRD analysis was carried out using an XPERT II: PANalytical XPert Pro MPD (Θ–Θ Diffractometer). The sample was positioned on a sample holder and exposed to Cu Kα radiation (8.04 keV and 1.5406 Å). Data were collected over a 5–20° range using Bragg–Brentano Θ/Θ‐diffractometer geometry with a 2θ step size of 0.007°. The XPS testing conditions for the wear tracks were consistent with those used for testing the COK‐47 powder. The surface morphology of the wear track was observed by SEM (Zeiss, Germany). Energy‐dispersive X‐ray Spectroscopy (EDS, Oxford Instruments, UK) was utilized to analyze the surface composition of the wear track. Transition electron microscopy (TEM, FEI TECNAI F20) was used to image the structure of the formed tribofilms. EDS inside the transmission electron microscope was performed using an EDAX‐AMETEK Apollo XLTW SDD system. For TEM investigation, a thin lamella was prepared by ThermoFisher Scios II Focused Ion Beam (FIB). The TEM lamellae were ≈30 × 10 µm^2^, with a thickness of <100 nm at the regions of interest. Selected area electron diffraction (SAED) patterns were used for phase analysis. Additional high‐resolution‐TEM (HR‐TEM) imaging was performed for phase analysis.

### Computational Simulation Methods

Spin‐polarized DFT calculations were carried out as implemented in version 7.2 of the Quantum ESPRESSO package,^[^ ^70^, ^71^, ^72^ ^]^ using the Perdew–Burke–Ernzerhof (PBE) parametrization for exchange and correlation functional.^[^ ^73^ ^]^ Dispersion corrections (DFT‐D2NG) were added to count for van der Waals interactions. The D2NG scheme is a modification of the Grimme‐D2 parametrization,^[^ ^74^ ^]^ in which the C6 and R0 coefficients of titanium are replaced with those of the preceding noble gas (i.e., Ar), according to a previous benchmarking made by some of the authors.^[^ ^75^ ^]^ The electronic wave functions (charge density) were expanded on a plane‐waves basis set, truncated with a cut‐off of 50 Ry (400 Ry). Default thresholds on the total energy (10−4 Ry) and forces (10−3 Ry Bohr^−1^) were adopted as relaxation thresholds. A Gaussian smearing of 0.02 Ry was used for describing the electronic state occupation around the Fermi level.

## Conflict of Interest

The authors declare no conflict of interest.

## Supporting information



Supporting Information

## Data Availability

The data that support the findings of this study are available from the corresponding author upon reasonable request.
