# Supplementary material for: Advanced Solid Lubrication with COK‐47: Mechanistic Insights on the Role of Water and Performance Evaluation
Source: Adv Sci (Weinh). 2025 Jan 13;12(17):2415268. doi: 10.1002/advs.202415268 (PMC12061247; doi:10.1002/advs.202415268)
Supplement: Supplementary file 1 — Supporting Information [file ADVS-12-2415268-s001.docx]

Advanced Solid Lubrication with COK-47: Mechanistic Insights on the Role of Water and Performance Evaluation

*Hanglin Li, Xudong Sui***, Pablo Ayala*, Edoardo Marquis, Hannah Rabl, Adrian Ertl, Pierluigi Bilotto, Yazhuo Shang, Jiusheng Li, Lu Xu, Maria Clelia Righi, Dominik Eder, Carsten Gachot**

H. Li, X. Sui, P. Bilotto, C. Gachot

Institute for Engineering Design and Product Development

Research Unit Tribology E307-05

TU Wien, Vienna, 1060, Austria

E-mail: xudong.sui@tuwien.ac.at carsten.gachot@tuwien.ac.at

L. Xu

State Key Laboratory of Solid Lubrication

Lanzhou Institute of Chemical Physics, Chinese Academy of Sciences,

Lanzhou, 730000, China

H. Li, J. Li

Laboratory for Advanced Lubricating Materials

Shanghai Advanced Research Institute, Chinese Academy of Sciences

Shanghai, 201210, China

P. Ayala, H. Rabl, A. Ertl, D. Eder

Institute of Materials Chemistry

TU Wien, Vienna, 1060, Austria

E-mail: pablo.ayala@tuwien.ac.at

Y. Shang

Key Laboratory for Advanced Materials

School of Chemistry and Molecular Engineering

East China University of Science and Technology

Shanghai, 200237, China

E. Marquis, M. C. Righi

Department of Physics and Astronomy “Augusto Righi”

Alma Mater Studiorum-University of Bologna

Bologna, 40127, Italy


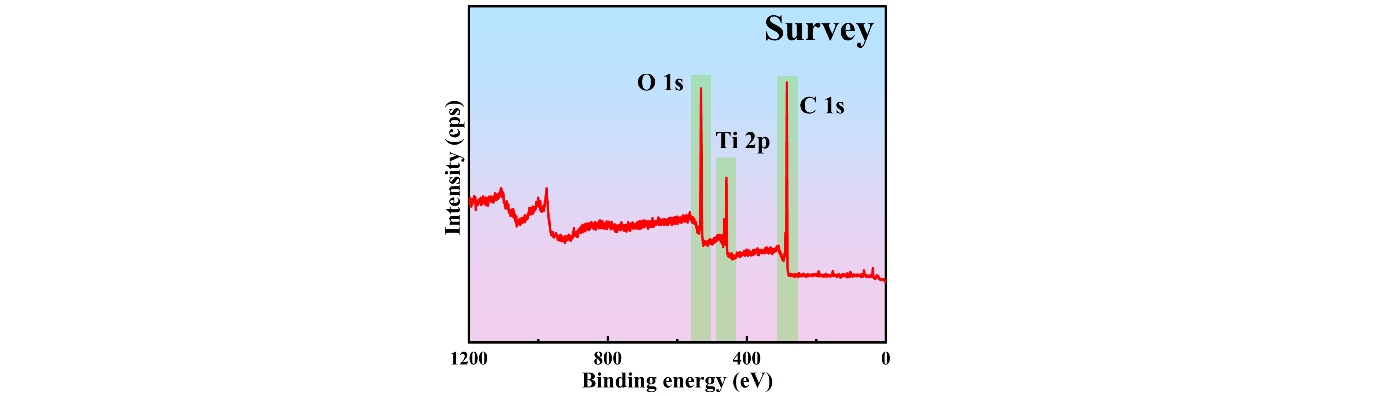


**Figure S1**. XPS survey spectrum of COK-47 powder.


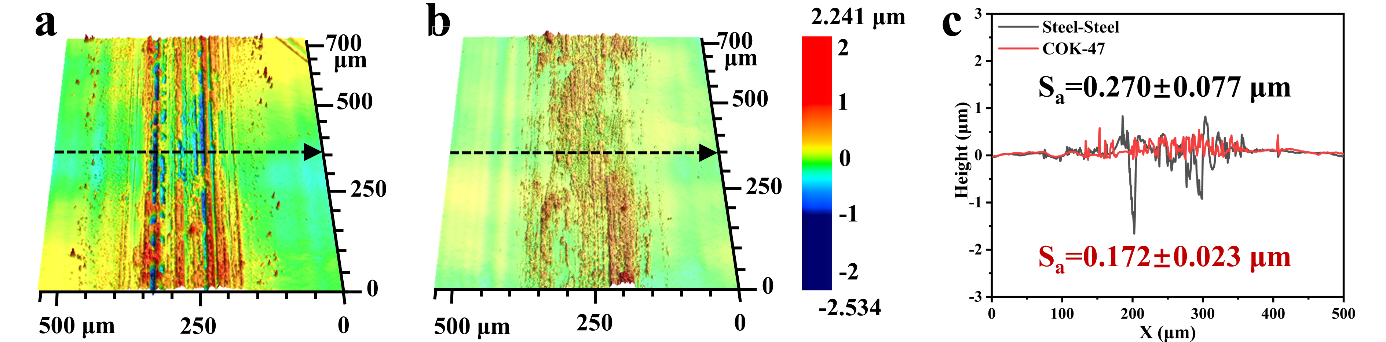


**Figure S2**. Tribological performance of COK-47 under steel-on-steel friction pairs. (a) and (b) are 3D profile images of without and with COK-47, respectively. (c) The cross-sectional height plot of the black dashed line in (a) and (b) shows the roughness of two wear tracks.

As the load increased, the width of the wear tracks increased, and some deeper grooves appeared (**Figure S3**). In addition, it was found that large colorful tribofilms were formed at the contact area after the low load tests (**Figure S3a, b**). Even after high-load testing, colorful films remained visible at the boundaries between the wear tracks and the untouched substrate. (**Figure S3d, e**). Therefore, it is speculated that COK-47 showed a good friction-reducing effect because of the formation of colorful tribofilm under friction, and this tribofilm will be consumed gradually under applied load.


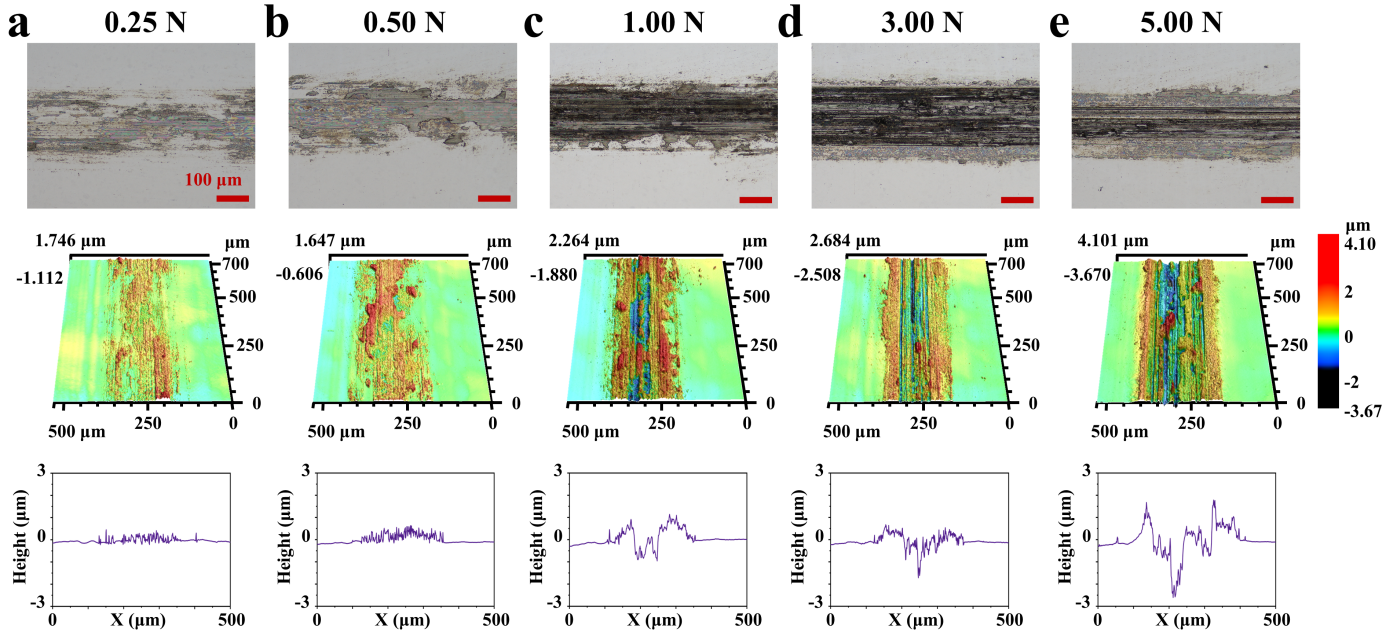


**Figure S3**. Tribological performance of COK-47 under different loads. (a) COF versus time under COK-47 powder with different loads. (b)-(f) are optical images, 3D profiles, and cross-sectional height plots in the center of the wear tracks under 0.25, 0.50, 1.00, 3.00, 5.00 N conditions, respectively.


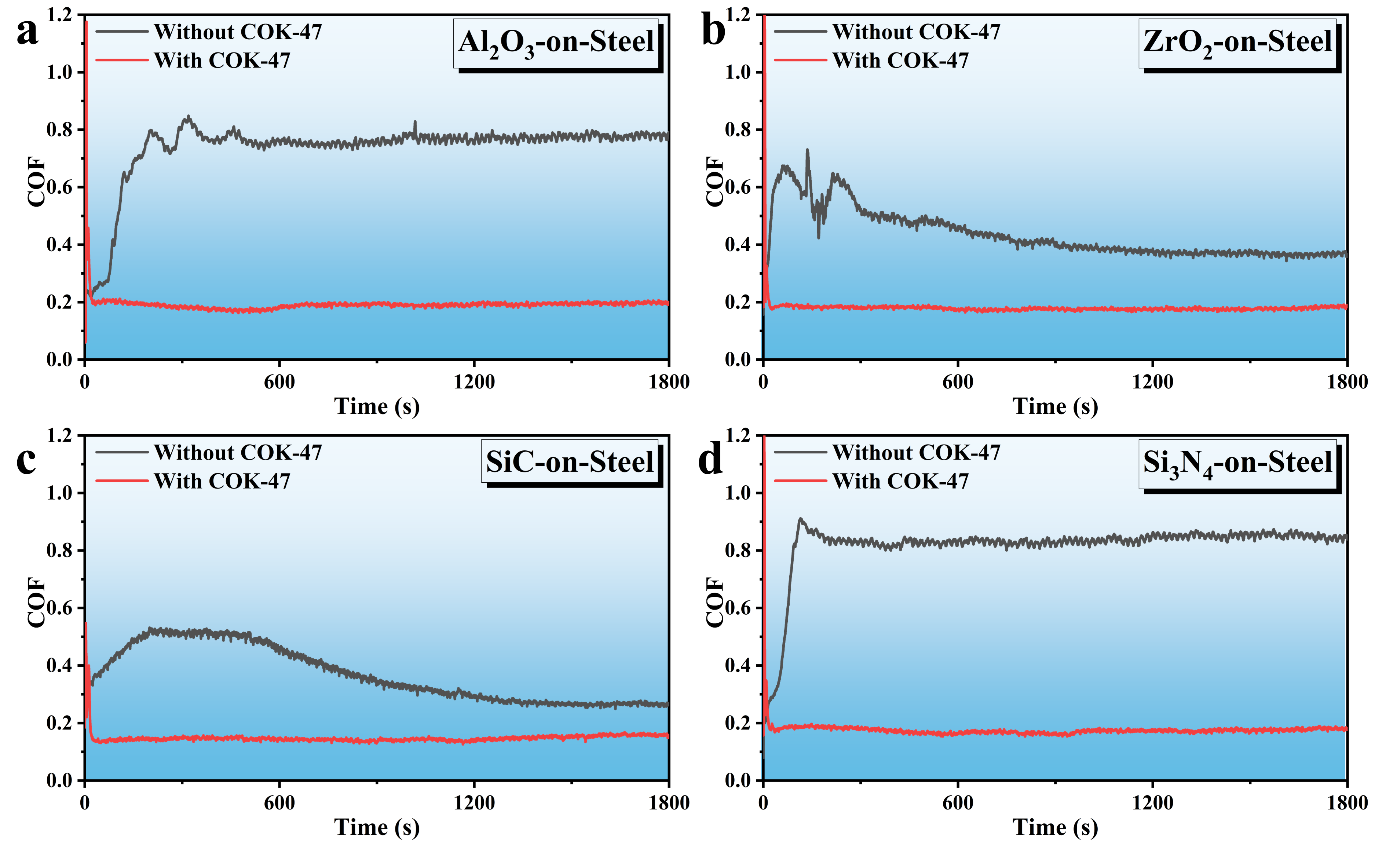


**Figure S4.** COF curves for COK-47 under different counter bodies. (a) alumina-, (b) zirconia-, (c) silicon carbide-, and (d) silicon nitride-on-steel.


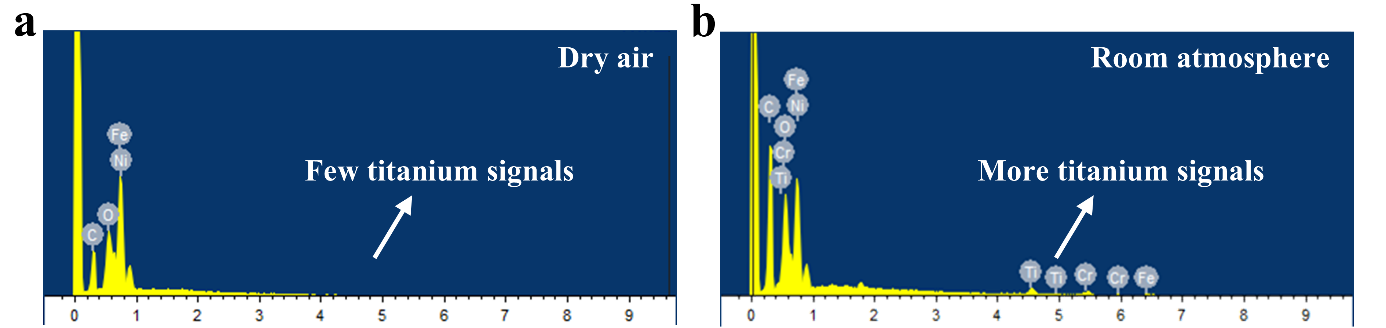


**Figure S5**. EDS elements spectra from the wear tracks under (a) dry air and (b) room atmosphere. Comparing EDS mapping results shows that there are more Ti and less iron signals. The tribofilm generated at optimal humidity is larger and denser. It is indicated that the Ti-containing tribofilm covered the substrate, reducing direct contact in the contact zone.

Additional tribological tests under different humidity conditions are exhibited in **Figure S6**. The results show that at low humidity, prominent dark areas appeared on the wear track with minimal tribofilm coverage (**Figure S6a, b**). As humidity increased, the dark areas gradually diminished, and the colorful tribofilm coverage expanded. The tribofilm was most continuous under room atmosphere (39.7-41.2% RH; **Figure S6e**). At higher humidity levels, the colorful tribofilm became partially consumed and discontinuous, but a distinct lubricating film persisted (**Figure S6f, g**). These findings highlight the critical role of appropriate environmental moisture in tribofilm formation.


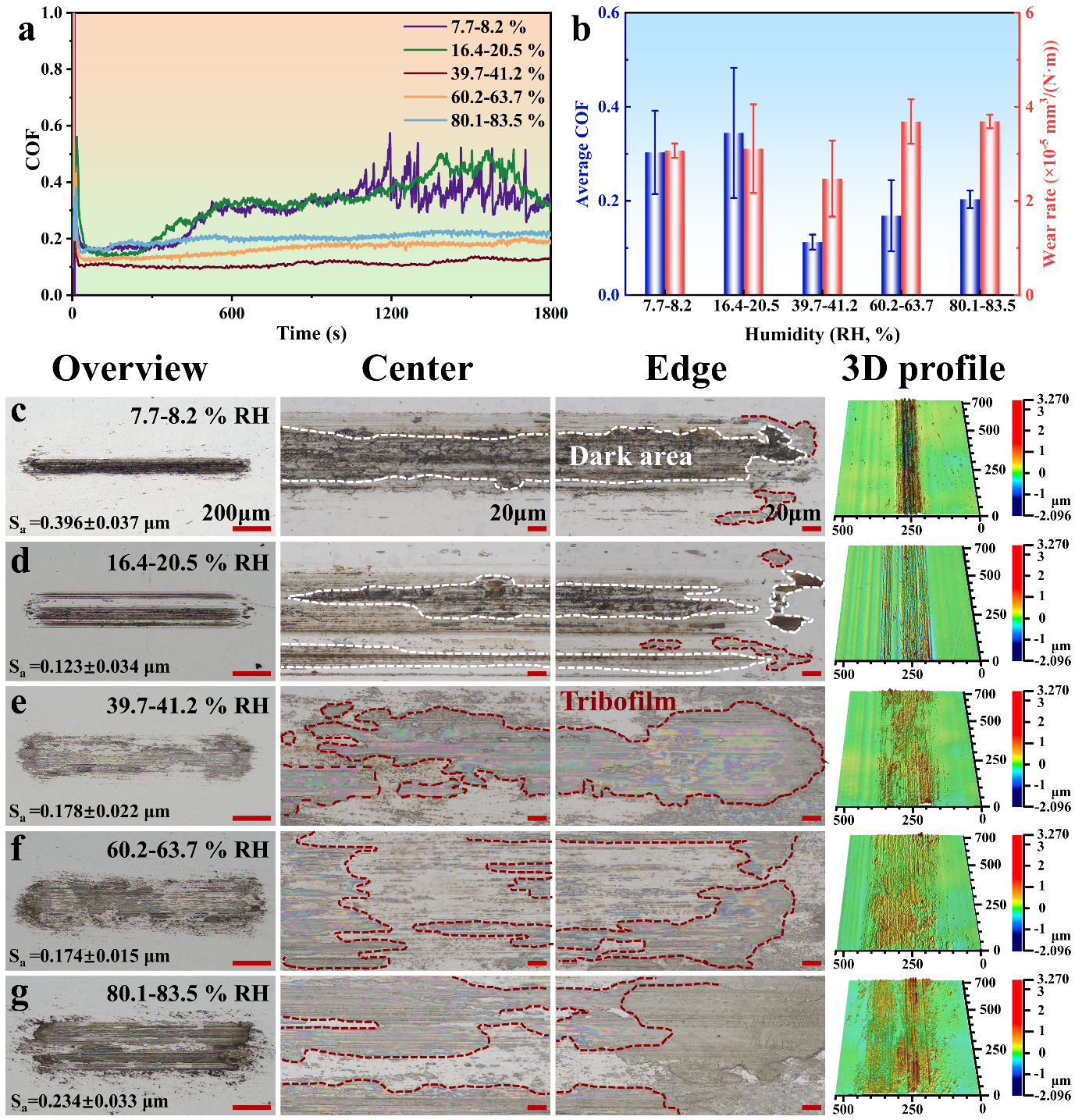


**Figure S6.** Tribological performance of COK-47 in different humidity ranges. (a) COF curves versus time under different humidity ranges. (b) Average COF and wear rate in different humidity ranges. Optical images, surface roughness, and 3D profile images of the wear tracks. (c) 7.7-8.2% RH, (d) 16.4-20.5% RH, (e) 39.7-41.2% RH, (f) 60.2-63.7% RH, and (g) 80.1-83.5% RH. The red dashed line indicated the colorful tribofilm and the white dashed line represented the area after severe wear, named the dark area.


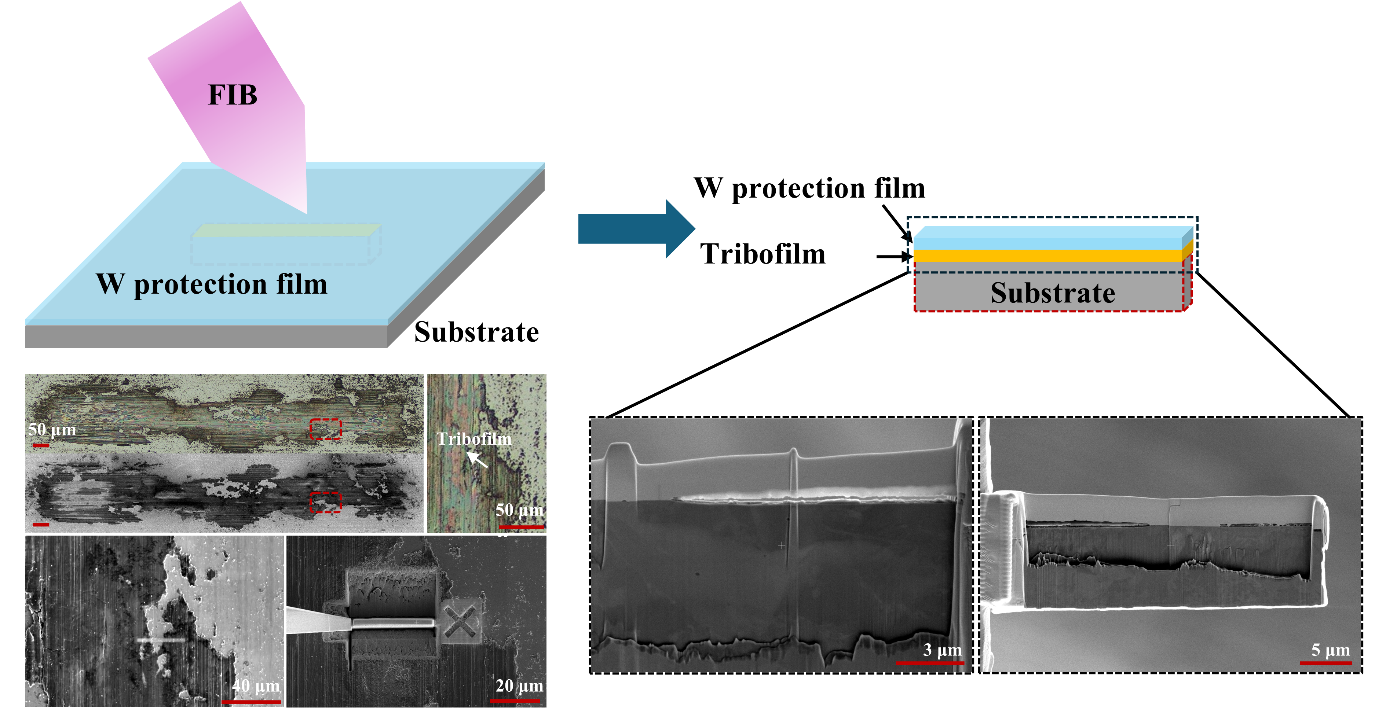


**Figure S7**. Schematic diagram of the FIB cutting process on the tribofilm on the wear track. The sample is the representative wear track after friction under room atmosphere, with high quality and continuous tribofilm.


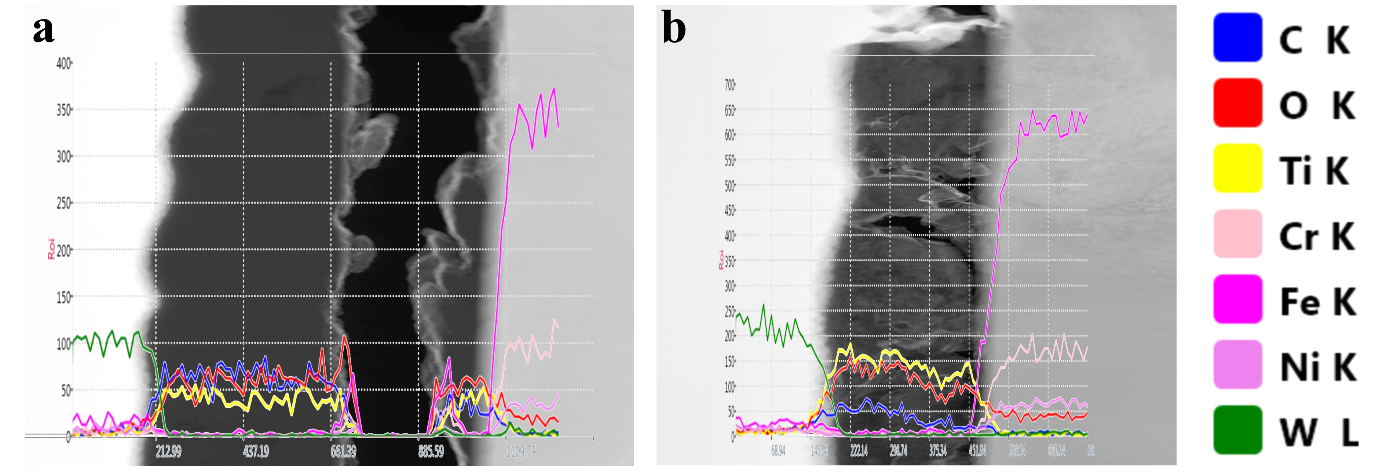


**Figure S8**. Linear elemental scanning mode of TEM on the lubrication film. It is demonstrated that the lubrication film is composed of C, O, and Ti.


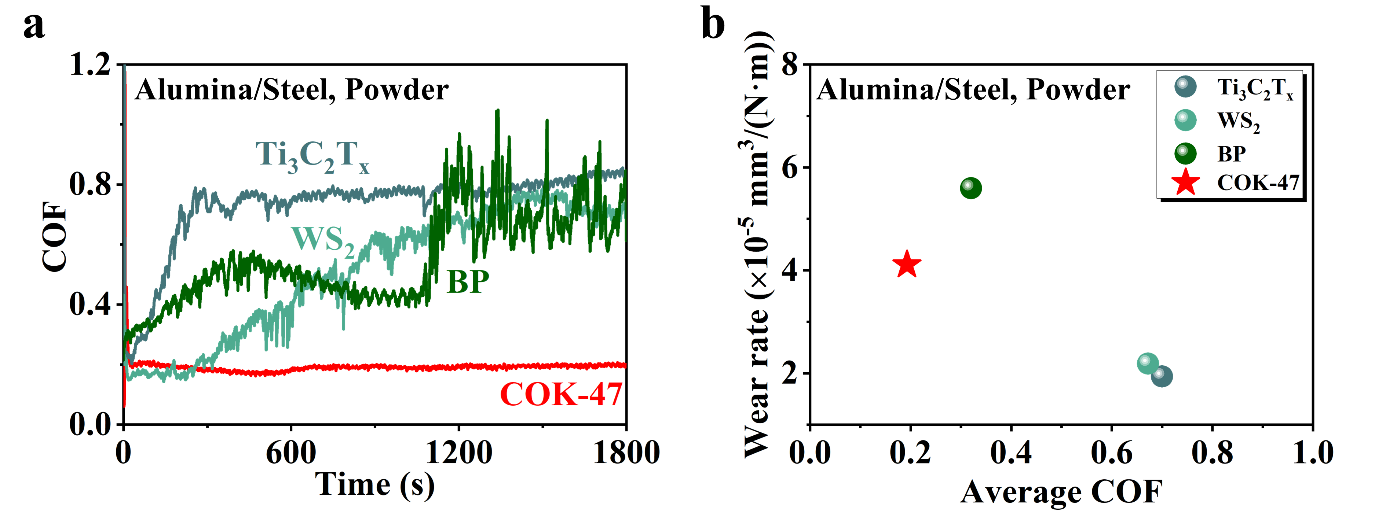


**Figure S9.** Comparison of tribological performance between COK-47 and other materials under alumina-on-steel sliding. (a) COF curves of different materials and COK-47. (b) Comparison of tribological performance (average COF and wear rate) between COK-47 and other widely studied materials.


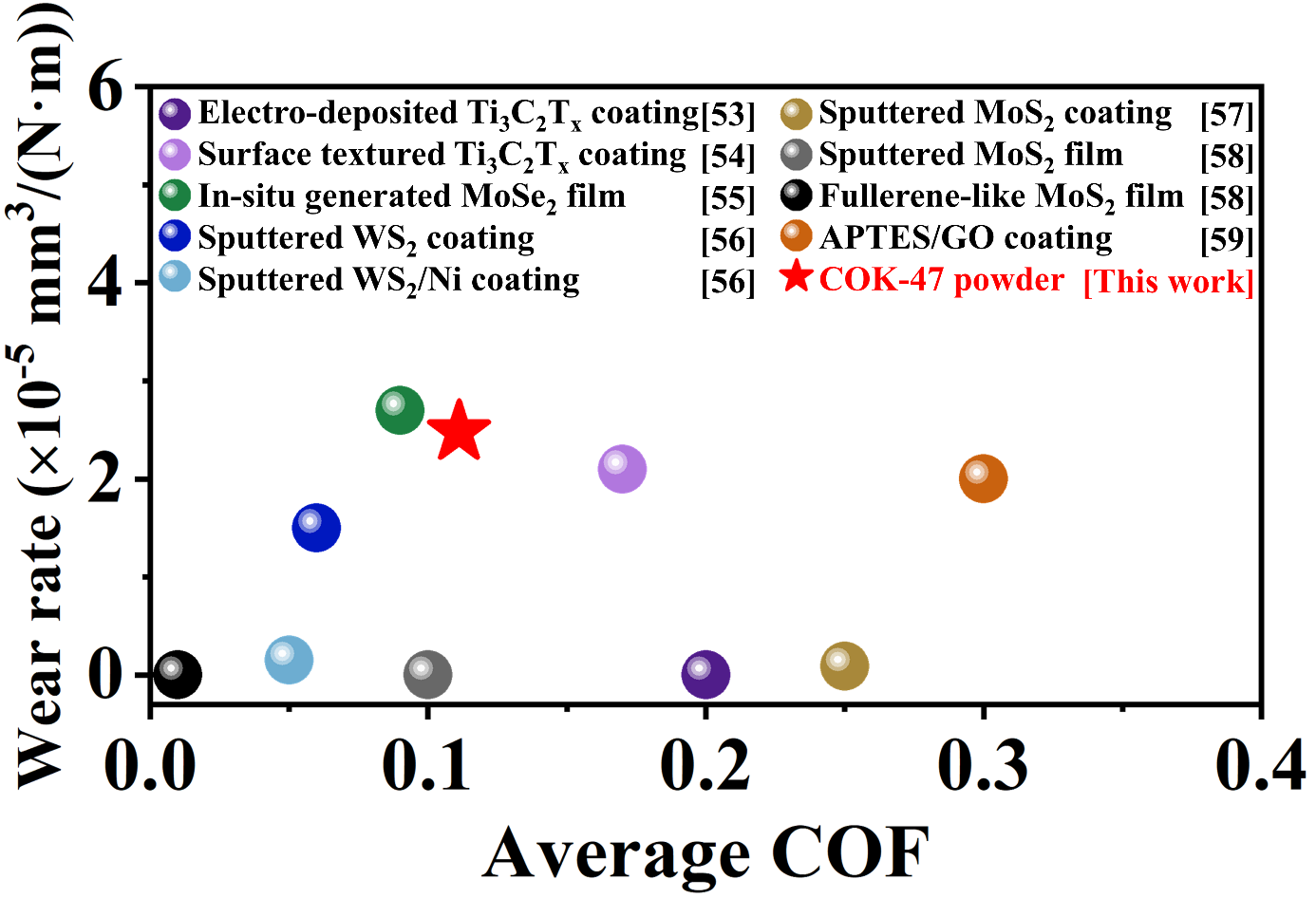


**Figure S10.** Comparison of tribological performance between COK-47 and other literatures.

**Table S1**. SEM-EDS elemental mapping results at the wear tracks under different conditions

| Elements | Dry air | | Room atmosphere | |
| --- | --- | --- | --- | --- |
|  | Weight% | Atomic% | Weight% | Atomic% |
| C | 8.61 | 27.22 | 15.30 | 40.39 |
| O | 6.52 | 15.47 | 6.84 | 13.55 |
| Ti | Below the instrument detection limit | | 4.20 | 9.59 |
| Fe and the others | 84.87 | 57.31 | 63.38 | 36.46 |
| Total | 100.00 | | 100.00 | |

**Table S2**. Raman characteristic peaks of pristine COK-47 (COK-47), fully friction-induced colorful tribofilm at room atmosphere with about 40% RH (Area 1 in **Figure 3d**), the gray area with incomplete friction at room atmosphere with about 40% RH (Area 2 in **Figure 3d**), less colorful tribofilm at dry air (Area 4 in **Figure 3d**), and the dark area with carbon and metal oxide resulting from insufficient lubrication at dry air (Area 5 in **Figure 3d**)

| COK-47 | Area 1 | Area 2 | Area 4 | Groups or Vibrations | Area 5 | Groups |
| --- | --- | --- | --- | --- | --- | --- |
| 1611 | 1610 | 1610 | 1618 | Strong aromatic ring stretching | 1612 | G peak |
|  | 1529 |  |  | -COO^—^/ Fe-COO^—^ | 1336 | D peak |
| 1456 | 1450 | 1453 | 1461 | Weak aromatic ring stretching | 669 | Fe_3_O_4_ |
| 1285 | 1282 | 1285 | 1295 | Inter aromatic ring stretching | 412 | Fe_2_O_3_ |
|  | 1204 |  |  | -COO^—^/ Fe-COO^—^ | 292 | Fe_3_O_4_ |
| 1156 | 1153 | 1154 | 1161 | Weak in plane deformation | 227 | Fe_2_O_3_ |
| 859 | 856 | 861 | 867 | Weak out of plane deformation | 161 | TiO_2_ |
| 631 | 629 | 628 | 638 | Weak aromatic system (C-C-C) | 140 | TiO_2_ |
| 416 | 418 | 412 | 422 | Ti-O |  |  |
| 133 | 131 | 130 | 139 | Ti-O |  |  |

**Table S3**. Ratios of the elements in tribofilm (**Figure 4d**)

| Elements | Weight% | Atomic% |
| --- | --- | --- |
| C K | 40.36 | 55.31 |
| O K | 35.49 | 36.51 |
| Ti K | 22.29 | 7.66 |
| Cr K | 0.36 | 0.11 |
| Fe K | 1.11 | 0.33 |
| Ni K | 0.23 | 0.06 |
| W L | 0.16 | 0.02 |
| Total | 100.00 | 100.00 |
